# Microbial Responses to Electric Field in Model Systems and Wastewater Applications: A Comprehensive Review

**DOI:** 10.1021/acsomega.6c01279

**Published:** 2026-06-25

**Authors:** Linda Štěpánková, Michaela Vranová, Petr Junga, Zuzana Tichá, Tomáš Vítěz

**Affiliations:** 1 Department of Agricultural, Food and Environmental Engineering, Faculty of AgriSciences, 309613 Mendel University in Brno, Zemědělská 1665/1, Brno 613 00, Czech Republic; 2 Department of Experimental Biology, Section of Microbiology, Faculty of Science, Masaryk University, Kotlářská 267/2, Brno 611 37, Czech Republic

## Abstract

The activated sludge process remains a cornerstone of biological wastewater treatment and relies on complex microbial consortia, including *Bacteria*, *Archaea*, and *Fungi*, for the degradation of organic pollutants and nutrient removal. The stability and efficiency of these microbial communities are influenced not only by wastewater composition but also by emerging technological interventions. The application of electric field (EF) has emerged as a promising and energy-efficient approach to improve treatment performance, modulate microbial behavior, and support bioresource recovery. Electric fields applied in wastewater treatment systems typically range from 5–20 A/m^2^ or 30–500 V/m, while pulsed electric fields may reach intensities up to 3.0–3.8 × 10^6^ V/m (40–200 μs), and have been reported to influence microbial activity and treatment performance. This review provides a comprehensive synthesis of EF-microorganism interactions in model systems and wastewater treatment applications, adopting a hierarchical framework that links molecular mechanisms to community-level outcomes. We discuss how cell wall architecture and physiological state determine microbial susceptibility to electroporation and other EF-induced phenomena, with particular emphasis on the structural and functional responses of microbial cell envelopes, including peptidoglycan, lipopolysaccharides, and S-layers. At the molecular and cellular levels, EFs induce ion fluxes, redox imbalance, gene regulation, membrane perturbation, cytoskeletal reorganization, and stress adaptation. At the population and community levels, EF exposure modulates quorum sensing, extracellular polymeric substance production, microbial adhesion, and biofilm structure, ultimately driving shifts in microbial community composition and functional potential through electrokinetic phenomena. Together, these multiscale effects influence key wastewater treatment outcomes, including pollutant degradation, sludge settleability, and bioenergy recovery. By integrating mechanisms across biological scales, this review provides a conceptual roadmap for the rational application of EF-based technologies in sustainable wastewater treatment systems.

## Graphical abstract

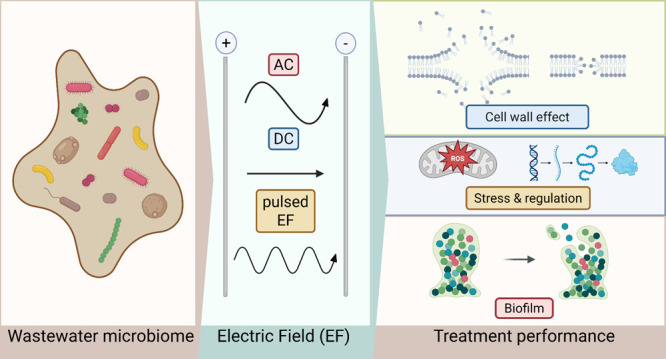

## 1 Introduction

Municipal wastewater treatment generates large amounts of sewage sludge worldwide, exceeding 13–14 million tons of dry solids annually in the European Union and more than 45 million tons globally. These amounts continue to increase due to urbanization, stricter wastewater treatment regulations, and the expansion of sewer networks.^1^ The activated sludge process remains the dominant biological treatment technology, relying on complex microbial consortia to degrade organic pollutants and remove nutrients from wastewater.^2^ The microbial communities driving activated sludge processes are composed primarily of *Bacteria*, *Archaea*, and *Fungi*, forming structured and functionally specialized consortia capable of organic matter degradation, nitrification/denitrification, and biological phosphorus removal.^3–7^ Community structure and function are strongly shaped by influent wastewater composition and operational conditions, with deterministic ecological processes contributing to the long-term stability and reproducibility of treatment performance.^8^ During treatment, several sludge streams are generated, including primary sludge from clarification and excess activated sludge from biological processes, which are subsequently stabilized by aerobic or anaerobic digestion to reduce organic load, pathogens, and odor prior to disposal or reuse.^9–11^

Beyond pollutant removal, activated sludge systems also substantially reduce the abundance of pathogenic microorganisms present in raw wastewater, although some free-living or resistant pathogens may persist after treatment.^12^ Maintaining microbial community stability is therefore essential, as disturbances such as sludge bulking or foaming can shift the community to a less desirable state, reducing treatment efficiency.^4^ Bioflocculation, a key feature of activated sludge, arises from the production of extracellular polymeric substances (EPS), primarily proteins and polysaccharides, which bind microbial cells and particles into stable flocs and strongly influence sludge settleability and process performance.^13,14^

In recent years, electric fields (EFs) have emerged as versatile physical tools for intensifying wastewater treatment processes. EF-based technologies are increasingly applied to enhance pollutant removal, improve sludge dewatering, suppress membrane fouling, and support bioresource recovery. For example, electro-dewatering accelerates solid–liquid separation through electrokinetic phenomena, enabling higher dry solids content with reduced energy demand compared with the conventional method.^15^ Pulsed electric field (PEF) technologies have demonstrated efficacy in reducing bacterial loads, including antibiotic-resistant strains, without generating harmful disinfection byproducts or promoting resistance development.^16^ Low-intensity or low-frequency EFs can also stimulate microbial metabolism, enhancing denitrification rates, modulating interspecies electron transfer, and improving methane production in bioenergy recovery systems.^17,18^ EF applications offer versatile, energy-efficient, and sustainable solutions for various challenges in wastewater treatment systems, similarly to other physical stimulation approaches such as low-intensity ultrasound or microwave pretreatment, which have also been shown to enhance microbial activity and treatment performance.^15,19,20^

EFs exert diverse and context-dependent effects on microorganisms, influencing survival, physiological activity, and ecological interactions. Both static and pulsed EFs can induce microbial inactivation through membrane permeabilization (electroporation), cell wall damage, and disruption of metabolic processes.^21–23^ The magnitude and direction of these effects depend on EF parameters including field strength, pulse duration, and waveform, as well as microbial traits such as cell wall architecture and physiological state.^24,25^ Importantly, EF exposure does not solely result in inactivation; under sublethal conditions, EF can modulate microbial growth, gene expression, biofilm formation, and community composition, with implications for wastewater treatment, bioremediation, and bioelectrochemical systems.^26–28^ Electrokinetic phenomena, including electrophoresis, electroosmosis, and dielectrophoresis, play critical roles in EF-mediated microbial responses, particularly in structured environments such as biofilms and sludge flocs. These processes govern the movement of charged cells, macromolecules, and fluids, thereby influencing microbial adhesion, biofilm morphology, mass transfer, and the transport of substrates or antimicrobial agents.^29^ At larger scales, EF application can reshape microbial communities and their functional potential, affecting treatment performance and system stability.

Despite increasing interest and successful pilot- and full-scale demonstrations, the mechanistic understanding of how EFs affect microorganisms across biological scales remains fragmented. Most studies focus on isolated effects, such as electroporation, oxidative stress, or biofilm disruption, without integrating these responses into a unified conceptual framework. A systematic synthesis linking molecular-level perturbations to cellular-, population-, and community-level outcomes is still lacking, particularly in the context of wastewater treatment systems. To address this gap, this Review adopts a hierarchical framework spanning multiple levels of microbial organization. We first describe molecular and intracellular events triggered by EF exposure, including ion fluxes, redox imbalance, and changes in gene regulation. These primary effects are then linked to cellular-level responses such as membrane perturbation, cytoskeletal rearrangements, stress adaptation, and apoptosis-like mechanisms. Subsequently, the review addresses population-level phenomena, including quorum-sensing modulation, extracellular polymeric substance (EPS) production, and alterations in adhesion and aggregation behavior. Finally, community-level consequences are discussed, focusing on shifts in microbial community composition, interspecific interactions, and functional potential in wastewater treatment systems. The conceptual framework used in this review, linking molecular-, cellular-, population-level, and community-level microbial responses to electric field exposure, is summarized in Figure 1.

**Figure 1:**
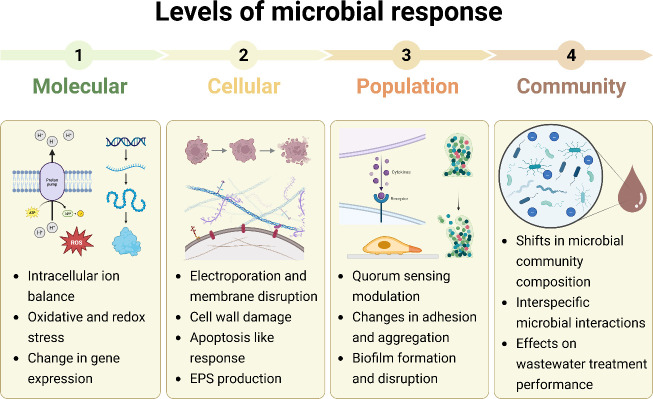
Roadmap of microbial responses to electric field exposure across molecular, cellular, population, and community levels.

## 2 Microbial Cell Structures and Their Susceptibility to EFs

The effectiveness of EF treatments depends on the type of microorganism: Gram-negative bacteria are typically more susceptible than Gram-positive bacteria and yeasts, probably because of differences in cell wall structure and thickness. Inactivation of more resistant species generally requires higher field intensities and a greater number of pulses.^24^ In addition, the physiological state of the cells, such as whether they are in the logarithmic or stationary growth phase, influences their susceptibility, with actively growing cells generally being more vulnerable.^30^ Although membrane permeabilization is commonly considered the primary mechanism, recent studies emphasize that cell wall architecture is also a direct target and that intracellular organization can also be disrupted.^31^ EFs can also trigger self-assembly behaviors in bacteria, with different species forming unique structures under alternating-current (AC) fields. This suggests that surface properties and cell wall characteristics play a key role in determining their response.^32^ Overall, the interaction between microbial cell structure and EF parameters is complex, yet the cell wall and membrane remain primary targets for effective inactivation of microbes.^31^ Table 1 summarizes the main microbial groups involved in wastewater treatment processes, together with their characteristic cell wall structures, EF sensitivity ranges, and proposed mechanisms of electric field action.

**Table 1:** Key Microbial Groups in the Wastewater Treatment Process

microbial group	Cell wall	EF sensitivity thresholds	proposed mechanisms of EF action	reference
*Pseudomonadota* (formerly *Proteobacteria*)	G-	high sensitivity	membrane destabilization, LPS disruption	^33–35^
*Bacteroidota* (formerly *Bacteroidetes*)	G-	high sensitivity	membrane permeabilization, oxidative stress	^34–37^
*Bacillota* (formerly *Firmicutes*)	G+	low sensitivity	resistance due to thick peptidoglycan, membrane permeabilization	^6,35,38^
*Actinomycetota* (formerly *Actinobacteria*)	G+, mycelium production	low sensitivity	resistance due to thick peptidoglycan, high stability filamentous structure	^6,35,39–41^
organohalide-respiring bacteria (OHRB)	peptidoglycan	moderate sensitivity	membrane permeabilization, oxidative stress	^42–44^
methanogenic archaea	pseudomurein, methanochondroitin S-layer	high sensitivity	extracellular electron transfer, membrane polarization, electrically induced charge redistribution	^45–49^
*Ascomycota*	chitin	moderate sensitivity	membrane permeabilization, enhanced enzyme susceptibility	^50–53^

### 2.1 Peptidoglycan

Peptidoglycan is a large, reticular polymer that forms an important part of the cell wall in most bacteria, supporting the structure and helping the cell to maintain its shape while protecting it from internal pressure and environmental threats.^54^ The saccharide component consists of alternating residues of *N*-acetylglucosamine (NAG) and *N*-acetylmuramic acid (NAM), linked by β-(1,4)-glycosidic bonds. A peptide chain composed of three to five amino acids is bound to the NAM, which enables cross-linking of the polymer.^55^ Due to the limited availability of studies directly conducted in wastewater systems, insights into the effects of EFs on peptidoglycan are often derived from studies on model microorganisms and analogous systems. Although these findings provide useful mechanistic hypotheses, their direct applicability to wastewater treatment environments remains uncertain because microbial cells in activated sludge are exposed to EFs under highly complex physicochemical and ecological conditions. Studies on model systems suggest that EFs can influence glycosidic bond cleavage by stabilizing or destabilizing reaction intermediates, particularly oxocarbenium ions, and by modulating the activation energy required for bond cleavage. In enzymatic systems, internal EFs generated by the protein environment have been shown to facilitate glycosidic bond cleavage by charge orientation and promotion of nucleophilic attack.^56^ The effects of different EF types on peptidoglycan vary significantly. PEF induces direct structural disruption through electroporation, leading to increased permeability and potential damage to peptidoglycan-associated structures.^57^ AC EF induces dynamic structural fluctuations in peptide assemblies by applying oscillating forces that can disrupt or loosen secondary structures. DC EF promotes more stable alignment of charged residues, leading to structural stabilization and controlled organization of peptide assemblies.^58^ Overall, most available evidence on EF-induced glycosidic bond cleavage, protein conformational changes, and peptide assembly originates from simplified systems, including purified biomolecules, isolated proteins, artificial membranes, molecular simulations, and pure microbial cultures. Real wastewater and activated sludge matrices are substantially more complex, containing heterogeneous microbial communities, EPS, suspended solids, ions, organic matter, and biofilm structures that may alter local electric field distribution and microbial responses.

### 2.2 Lipopolysaccharides

Lipopolysaccharides (LPSs) are a major component of the outer cell wall of Gram-negative bacteria. Under the influence of an EF, reorganization and separation of membrane components, particularly lipids, can be induced, potentially altering the distribution and function of LPS in the cell wall by promoting lateral remixing and phase separation within the membrane structure.^59^ Studies on model membranes suggest that EF exposure may modulate the friction and mechanical properties of lipid bilayers, potentially affecting the stability and protective function of the LPS layer.^60^

### 2.3 S-layer

S-layers, or surface layers, are crystalline, monomolecular assemblies of proteins or glycoproteins that form a regular, two-dimensional lattice on the outermost surface of many bacteria and nearly all archaea.^61^ Proteins in S-layers are mainly held together by noncovalent interactions, such as hydrogen bonds, ionic interactions, and hydrophobic forces.^62^ An external EF may enhance or weaken these interactions depending on their orientation and strength, often by promoting charge transfer, increasing polarization, or modifying the distribution of electron density between interacting partners.^63^

## 3 Electroporation and Membrane Disruption Mechanisms (Fundamentals of the Effect of EFs on Microorganisms and Sludge)

### 3.1 Types of EFs Used in Wastewater Treatment

EFs can be applied in wastewater treatment in several forms, including pulsed, alternating, and direct fields, each operating through distinct mechanisms to support pollutant removal, disinfection, and overall process intensification. The selection of a specific field type depends on the nature of the contaminants, treatment objectives, and operational constraints. The principal types of EFs applied in wastewater treatment, their mechanisms of action, and associated process benefits are summarized in Table 2.

**Table 2:** Types of EFs Used in Wastewater Treatment

EF type	mechanism	treatment function	reference
alternating current (AC)	AC EF enhance nutrient transport and mass transfer in microbial systems by promoting ionic migration, inducing oscillatory electroosmotic flows, and altering membrane permeability, thereby increasing substrate availability and microbial metabolic activity	boosts biological denitrification, reduces energy use	^18,28,64^
direct current (DC)	DC EF can modify membrane potential and metabolic activity, thereby enhance pollutant degradation and support electroactive bacteria.	heavy metal removal, desalination, pollutant aggregation	^34,65,66^
pulsed electric field (PEF)	PEF affects microorganisms by disrupting cell membranes through short high-intensity pulses (electroporation), leading to loss of cellular integrity and subsequent inactivation or cell death.	disinfection, sludge treatment, enhanced biogas production	^16,67,68^

### 3.2 Electroporation

Electroporation is a process in which an EF induces the formation of transient pores in the lipid bilayer of the cytoplasmic membrane, allowing substances to enter or exit the cell. The process can be reversible, with the membrane resealing and the cell surviving, or irreversible, leading to cell death if the damage is too extensive.^69^ When microorganisms are exposed to an EF, the transmembrane voltage of the cell membrane increases. If this voltage exceeds a critical threshold, typically around 0.7 to 1.15 V, then nanometer-sized pores form in the cell membrane.^23^ The principle of pore formation in the membrane lies in the alteration of the spatial arrangement of phospholipids, see Figure 2 for details. When the transmembrane potential exceeds a critical threshold, the polar heads of the phospholipids rotate, leading to the formation of a pore.^70^ Among taxa, reported electroporation thresholds, the minimum EF strength required to permeabilize cell membranes, vary between taxa due to differences in cell wall structure, membrane composition, and physiological properties, as summarized in Table 3.^71^

**Table 3:** Limit Values for Groups of Microorganisms when Electroporation Occurs

microbial group	cell wall structure	electroporation threshold	reference
G+ bacteria	thick peptidoglycan layer	1.0 × 10^6^–2.0 × 10^6^ V/m	^72–75^
G- bacteria	thin peptidoglycan layer, outer membrane	3.5 × 10^5^–2.0 × 10^6^ V/m	^71,76,77^
fungi	thick cell wall, glucans, mannans, chitin	2.0 × 10^5^–1.0 × 10^6^ V/m	^78–81^
archaea	S-layer pseudomurein	3.6 × 10^5^–5.6 × 10^5^ V/m	^71,82,83^
protozoa	no cell wall	2.0 × 10^5^–2.5 × 10^5^ V/m	^84,85^
algae	cellulose, polysaccharides	0.5 × 10^5^–7.0 × 10^5^ V/m	^86,87^

**Figure 2:**
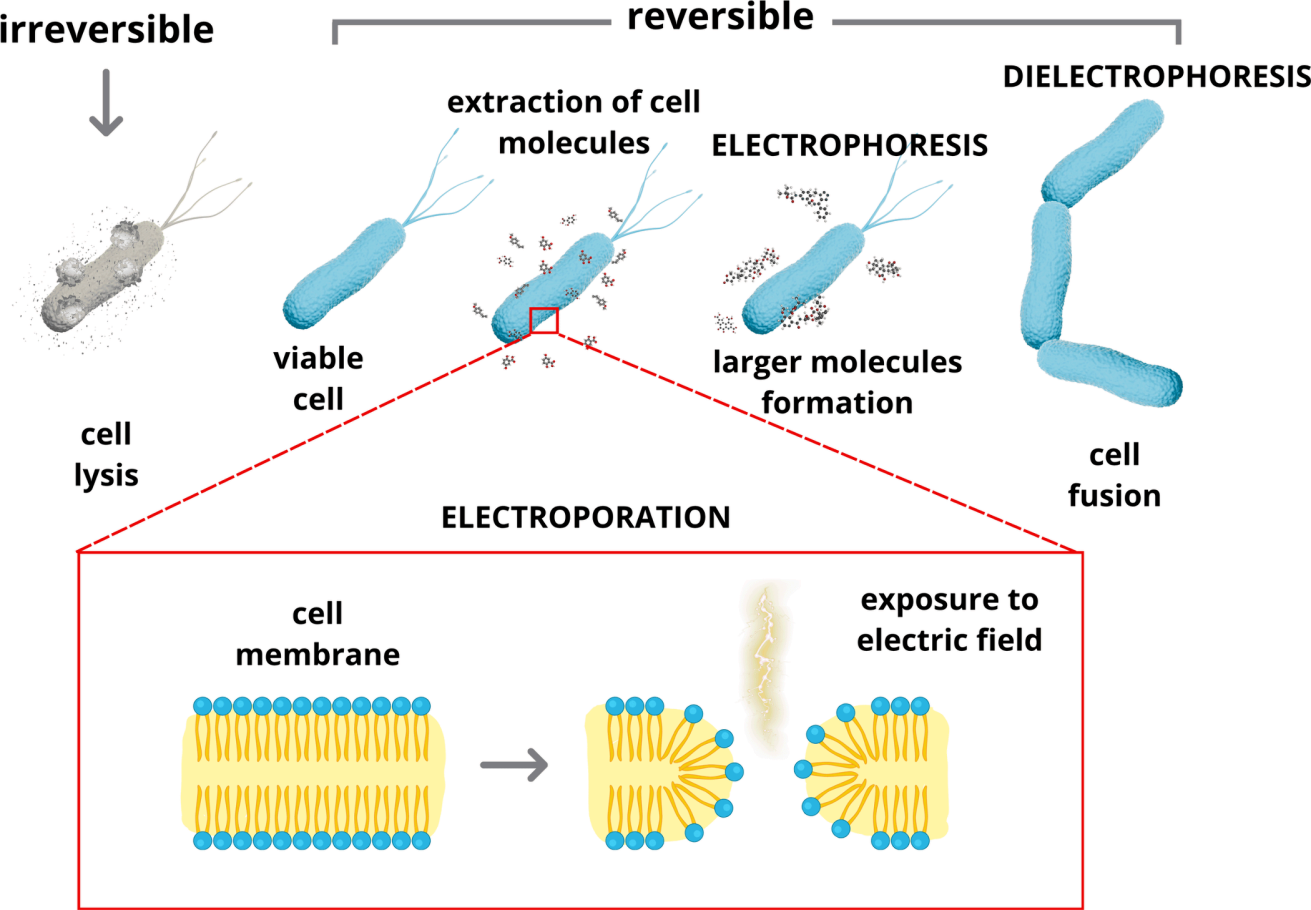
Overview of electroporation effects on cells, illustrating irreversible cell lysis and reversible outcomes including molecular extraction, electrophoretic transport, and dielectrophoresis induced cell fusion. Corresponding electroporation thresholds range from 0.5 × 10^5^ to 2.0 × 10^6^ V/m depending on the microbial group (see Table 3 for details).

### 3.3 Electrophoresis

Electrophoresis in cells refers to the movement of charged molecules or even whole cells under the influence of an EF. On the cellular membrane, bio-EFs can cause charged components, such as proteins and lipids, to migrate along the membrane surface, leading to their separation and influencing cell function and signaling.^88^ The principal mechanisms of EF-induced membrane disruption, including reversible and irreversible electroporation as well as electrophoretic and dielectrophoretic effects, are schematically illustrated in Figure 2.

## 4 Molecular and Intracellular Mechanisms Induced by Electric Fields

At the most fundamental level, electric fields first perturb microorganisms through molecular and intracellular processes, which represent the primary interface between the physical stimulus and the biological response. These early events set the stage for downstream cellular, population-level, and community-level effects observed in wastewater treatment systems. Intracellular ion fluxes and homeostasis were disrupted.

### 4.1 Intracellular Ion Balance

Intracellular ion balance in bacterial cells can be influenced by an EF, especially Ca^2+^ and K^+^ levels. During processes such as electroporation, the increased membrane permeability allows rapid ion movement across the cell membrane, altering their intracellular concentrations.^89,90^ Pulsed EF can be used to increase the accumulation of Ca^2+^ inside bacterial cells, as shown in applications such as probiotic enrichment. The efficiency of Ca^2+^ uptake depends on the strength and duration of the EF.^91^ Short electrical pulses can cause K^+^ to exit bacterial cells through specific channels, leading to changes in the membrane potential. The direction and magnitude of this effect can depend on the physiological state of the cell and the presence of certain inhibitors or environmental conditions. Loss of ion homeostasis, especially K^+^ leakage, is linked to bacterial inactivation and loss of viability.^89^

### 4.2 Oxidative and Redox Stress at the Molecular Level

EFs can indeed contribute to oxidative stress in microorganisms by promoting the formation of reactive oxygen species (ROS), which leads to oxidative damage and triggers antioxidant responses. Exposure to various types of EFs, including PEF, nanosecond pulsed EF (nsPEF), and high-voltage electrostatic fields, has been shown to increase intracellular and extracellular ROS levels, such as hydrogen peroxide and superoxide anions, in both bacteria and eukaryotic cells.^92–94^ This ROS accumulation can damage cellular components, including membranes, proteins, and DNA, ultimately resulting in cell death or apoptosis.^95^

The oxidative stress induced by EFs also alters the expression of genes related to antioxidant defense, such as the genes encoding superoxide dismutase and catalase, and can deplete antioxidant molecules like glutathione.^93^ There is also an effect on intracellular signaling pathways that detect oxidative stress and trigger a defensive response. The application of an EF can cause the dissociation of the bond between the antioxidant protein TRX and its inhibitor, TXNIP. This activates TRX, enabling it to more effectively protect the cell from oxidative stress.^96^

### 4.3 EF-Driven Gene Expression and Regulatory Responses

EFs can also influence gene expression in relation to cellular stress. PEF likely induces mild abiotic stress, which activates defense pathways similar to those triggered by pathogen attack, thereby promoting the biosynthesis of protective compounds.^97^

PEF also influences gene expression related to sugar metabolism, EPS biosynthesis, and the stress response. Activation of metabolic pathways associated with cell wall modification was also confirmed.^98^ It was also found that PEF significantly increases the expression of genes involved in the oxidative stress response, particularly GSH1, GLR1, SOD1, and SOD2, which are associated with the synthesis and utilization of glutathione. At the same time, the theory that PEF induces thermal stress was not supported.^99^

### 4.4 Perturbation of Signal Transduction Pathways

EFs can strongly influence signaling impact signal transduction processes in microorganisms as well as in other cell types. Studies have shown that these fields can alter cellular behaviors such as movement, growth, and specialization by affecting various signaling mechanisms, including ion channel activity, membrane receptor function, and internal signaling cascades.^100^

Collectively, these molecular and intracellular perturbations represent the primary responses of microorganisms to electric field exposure and form the mechanistic basis for subsequent structural, physiological, and functional changes at the cellular level.

## 5 Cellular-Level Responses to Electric Fields

Molecular and intracellular perturbations induced by electric fields subsequently manifest as structural and functional changes at the cellular level. These responses affect membrane integrity, cytoskeletal organization, stress survival strategies, and cell fate, thereby shaping the microbial viability and functionality under EF exposure.

### 5.1 Effects on Cytoskeleton and Cellular Architecture

EFs can influence bacterial cell division by interacting with the protein FtsZ, which is essential for the formation of the division ring (Z-ring) that orchestrates cytokinesis. FtsZ has been shown to generate its own electrical oscillations as it assembles into filaments and sheets, suggesting that it has intrinsic electrical properties that may respond to external EFs.^101^ If external EFs alter the electrical behavior of FtsZ, they may disrupt its polymerization, its treadmilling dynamics, or its interactions with partner proteins, potentially impairing the precise timing and localization of cell division.^101,102^ While direct studies on the effects of EFs on MreB are limited, it is known that MreB polymerization and filament stability are highly sensitive to ionic conditions, including concentrations of monovalent and divalent cations like potassium, sodium, and calcium.^103^

EF can significantly influence the structure and function of proteins, including bacterial proteins like crescentin. Research shows that exposure to EFs may induce conformational changes in proteins, such as unfolding, stretching, or transitions from helical to random coil structures, especially at higher field strengths, which weakens their structural stability and alters their mechanical properties.^104^ These changes are driven by the field’s effect on intraprotein hydrogen bonds and the protein’s dipole moment, which may lead to rapid and sometimes reversible structural transitions.^105^ Electrical exposure can also influence protein aggregation and gelation, as demonstrated in studies on other filamentous proteins. These effects may alter the assembly and stability of crescentin, potentially disrupting its function as a cytoskeletal protein.^106^

### 5.2 Stress Survival Strategies and EPS Production

Exposure to EFs significantly affects EPS production, composition, and distribution in wastewater microorganisms. The response depends not only on electrochemical effects but also on EF strength, exposure time, microbial species, and reactor conditions.^107–109^ Consequently, no universal effect of EFs on EPS has yet been established. Weak EFs may stimulate the protective function of EPS as an adaptive microbial response, whereas stronger or prolonged EF exposure can induce excessive EPS production or EPS degradation, thereby disrupting the protective function of the biofilm matrix.^110–112^ The impact of EF strength is nonlinear: weak to moderate fields (e.g., 10–80 V/m) often reduce EPS content by stimulating the growth of EPS degrading bacteria or enhancing enzymatic breakdown.^113,114^ Microbial responses to EFs are species dependent, as *Bacteroidetes*, known for high proteinaceous EPS secretion, may be suppressed, whereas EPS degrading microorganisms, such as *Patescibacteria*, can become enriched under weak EFs.^107^ In membrane bioreactors (MBRs), applying a weak EF reduces EPS content by over 40%, alters microbial communities to favor EPS degrading bacteria, and enhances enzymatic activity, thereby mitigating membrane fouling and improving effluent quality with lower energy consumption.^115^ The EF also modifies EPS composition by decreasing proteins relative to polysaccharides, increasing negative charge and electrostatic repulsion between sludge and membranes, which reduces fouling.^116^ Moderate EF exposure may stimulate EPS biosynthesis in certain microorganisms. In a bioelectrochemical system, exposure of Pseudomonas sp. DGYH-12 to a microelectric field of 0.2 V increased EPS production from 33.36 to 39.38 mg/L (approximately 18%) while simultaneously enhancing phenanthrene degradation efficiency.^117^ Excessively strong EFs, however, may damage cell membranes, disrupt biofilm structure, and impair microbial activity. A schematic overview of EF-induced cellular stress responses, including membrane disruption, oxidative stress, gene regulation, and EPS overproduction, is shown in Figure 3.

**Figure 3:**
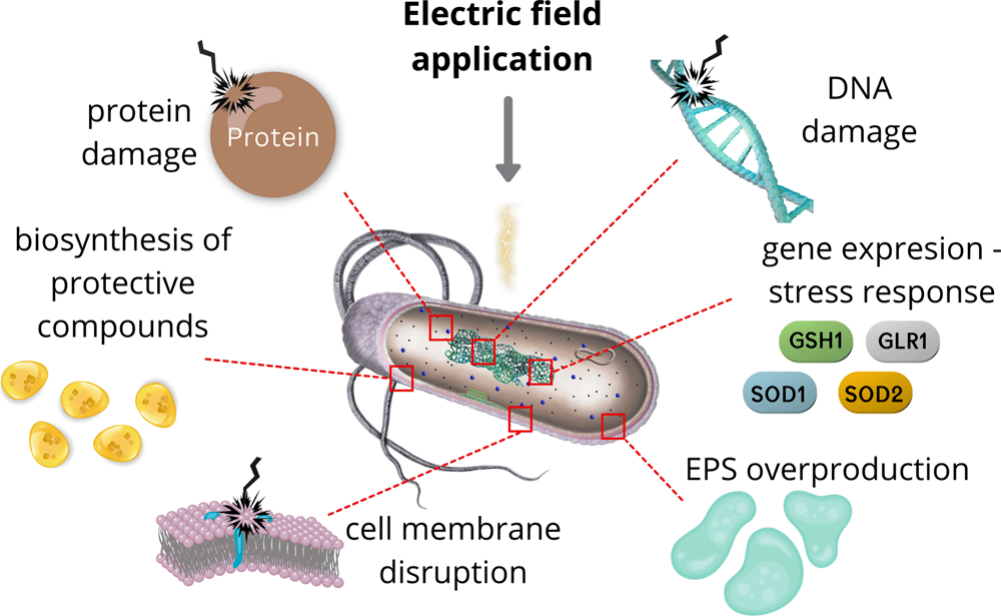
Cellular responses to electric field exposure include membrane disruption, protein and DNA damage, induction of stress response gene expression, biosynthesis of protective compounds, and enhanced EPS production. Stress response genes GSH1 and GLR1 (glutathione metabolism) and SOD1/SOD2 (superoxide detoxification) are upregulated in response to electric field induced oxidative and membrane damage. While oxidative stress, stress-related gene expression, and electroporation typically occur under PEF conditions in the range of 1.0 × 10^5^–8.0 × 10^6^ V/m, EPS production can be influenced by substantially lower electric field strengths, approximately 10–80 V/m.

### 5.3 Apoptosis-Like Response and Programmed Cell Death Mechanisms

Some research reports that EFs can induce features similar to apoptosis in bacteria, such as membrane potential loss, esterase activity reduction, and internal structural damage. However, these features are not identical to classical apoptosis in animal cells and may represent a combination of different cell death pathways.^118^ Studies using low-frequency, low-voltage AC found that only a small proportion of bacterial cells (about 10%) showed apoptotic markers, suggesting that apoptosis is not the main outcome under these conditions.^119,120^

These processes, including intracellular ion regulation, signal transduction pathways, and apoptosis-like responses, are closely linked to microbial communication, as they integrate electrical and chemical signaling systems such as quorum sensing and influence gene regulation.^121–124^

These cellular-level responses integrate molecular stress signals into altered microbial behavior and survival strategies, thereby governing population-level adaptations and shaping community processes under electric field exposure.

## 6 Population and Community-Level Effects of Electric Fields

Cellular responses to electric fields collectively influence microbial behavior beyond the single-cell level, giving rise to population-level adaptations and community restructuring. Through modulation of microbial communication, adhesion, biofilm formation, and interspecies interactions, EF exposure can ultimately reshape community composition and functional potential in wastewater treatment systems.

### 6.1 EF Effects on Microbial Communication and Quorum Sensing

Current knowledge on EF effects on quorum sensing (QS) and quorum quenching in biofilms is still limited. The application of an EF (5 A/m^2^) led to a reduction of QS molecules by up to 76% (especially AHL), indicating effective degradation.^125^ In addition, the EF generates reactive oxidants (e.g., chlorine or oxygen) that can directly degrade QS signaling molecules, a process known as ″quorum quenching″, which means the disruption or inhibition of microbial cell-to-cell communication (quorum sensing), thereby preventing coordinated behaviors such as biofilm formation or virulence expression.^126^

In contrast to their inhibitory effects, EF can also promote the activity of QS under certain conditions. For example, a low-voltage EF of 0.5 V in anaerobic membrane bioreactors activated QS genes in nonelectroactive bacteria, leading to increased biofilm formation and metabolic activity.^127^ Similarly, in a denitrifying microbial electrolysis cell, 0.8 V stimulation increased phenazine derivatives and AHL-mediated QS signaling in *Pseudomonas aeruginosa* PAO1, accompanied by strong upregulation of QS-related genes and metabolic reprogramming.^128^

Applying low auxiliary voltages (e.g., 0.5–1.1 V) in anaerobic bioreactors can also stimulate extracellular electron transfer between microorganisms, increasing metabolic activity and possibly influencing QS-regulated behaviors. Methanol was probably the electron carrier of methanotrophs and electroactive bacteria.^129^ Overall, the relationship between EF strength, exposure duration, and the shift between QS activation and quorum quenching remains largely uncharacterized due to the lack of systematic studies. A possible explanation for the observed biphasic response is that EF effects may promote oxidative degradation of QS signaling molecules, resulting in quorum quenching. In contrast, under different reactor conditions, EFs may stimulate microbial metabolism, extracellular electron transfer, and QS-related gene expression.

### 6.2 Changes in Bacterial Adhesion, Aggregation, and Biofilm Formation

An EF influences bacterial adhesion to surfaces by altering their surface charge and electrostatic interactions, which can either promote or inhibit biofilm formation.^130^ Electrokinetic phenomena influence bacterial deposition on surfaces; electrophoresis promotes adhesion, while electroosmosis limits biofilm adhesion to the surface.^29^ The inhibition of biofilm growth is primarily caused by dielectrophoresis during cell division, when a constriction forms between the daughter cells and causes the movement of macromolecules, which can disrupt the stability of the cell wall, the enzymatic processes of cell division, and the division apparatus (bacterial protein FtsZ).^131^ Electrochemical reactions at the electrodes generate reactive oxygen species that can disrupt the EPS matrix responsible for the cohesion of the biofilm or lead to cell death. In addition, this process can positively influence the presence of electroactive species (e.g., *Geobacter* and *Shewanella*).^132^ According to ref,^133^ the arrangement of electrodes also influences biofilm formation. The study shows that a vertical electrode configuration leads to greater cell accumulation and the development of a more compact biofilm. Low EFs can stimulate microbial activity, increase degradation rates, and enrich beneficial microbial groups, which support community stability and biofilm function.^28^ For example, Gram-positive bacteria (especially *Bacillota* and *Actinomycetota*) play a dual role in the biofilms of microbial fuel cells. Structurally, they contribute to the stabilization of the biofilm due to their thick cell wall, their resistance to stress, and their ability to secrete EPS. Functionally, they are usually not electroactive themselves, but they support electroactive bacteria through syntrophic interactions or substrate degradation.^134^

The EF increases the negative charge of the EPS, which increases the electrostatic repulsion between the EPS and the membrane surface, thereby reducing bacterial adhesion and biofilm formation.^126^ The distribution of EPS components within the biofilm changes with the electric potential and influences how cells interact with the electrode and with each other. This spatial arrangement can influence both the efficiency of electron transfer and cell protection.^135^ The ability of a biofilm to generate current is closely related to the redox capacity of its EPS. Biofilms with higher EPS redox activity (more proteins and humic substances) show greater current production, while those with less redox-active EPS produce less current.^136^

The ratio of protein and polysaccharide components is also influenced by the application of an EF. A lower potential leads to more redox-active proteins and fewer polysaccharides in EPS, which improves electron transfer.^135^ Higher potentials lead to more polysaccharide-rich, nonconductive EPS layers, which can act as barriers but can protect cells from electrical stress.^136^

Electrokinetic phenomena, which refer to the movement of charged particles, fluids, or cells under the influence of an electric field, including electrophoresis, electroosmosis, and dielectrophoresis, play an important role in the formation and manipulation of biofilms. Electrophoresis controls the migration of charged cells and particles, electroosmosis induces fluid flow within EPS, and dielectrophoresis affects the spatial distribution of cells in nonuniform EFs. The balance between these forces determines the strength of cell adhesion, the morphology of the biofilm, and the efficiency of transport of particles or antimicrobial agents into the biofilm.^29^

### 6.3 Population-Level Adaptation and Selection Pressures

In wastewater treatment systems, intermittent EFs caused shifts in microbial populations, while continuous fields maintained more stable communities, indicating that changes depend on the mode and intensity of EF application.^137^ Some studies also report enrichment of specific bacteria adapted to EF conditions without complete community replacement, suggesting a selective rather than irreversible effect.^138^ EF can enhance pollutant removal and enrich functional bacterial groups, such as *Pseudomonadota* and *Chloroflexota*, by promoting metabolic activities linked to biodegradation.^139^ Long-term EF exposure in membrane bioreactors improves pollutant degradation and shifts microbial populations by reducing biofilm-forming bacteria and promoting biopolymer-degrading genera, which also mitigates membrane fouling.^140^ Overall, EF application affects microbial diversity trends by modifying community structure, metabolic activity, and environmental conditions, with effects depending on field strength and duration.^141^

## 7 Shifts in Microbial Community Composition and Functional Potential

### 7.1 Interspecific Microbial Interactions under EF Exposure

EFs influence the interactions between electroactive and nonelectroactive microorganisms by modulating electron transfer processes and microbial metabolism. Electroactive microbes facilitate extracellular electron transfer through mechanisms such as cytochrome-based pathways, conductive protein filaments, and soluble electron shuttles, enabling direct or indirect electron exchange with electrodes or other cells.^142,143^ Application of EF can enhance syntrophic metabolism by promoting direct interspecies electron transfer, enriching electroactive species, and optimizing metabolic pathways that produce methane and other products.^144,145^ Intermittent EF has been shown to regulate microbial community composition, favoring fermentative bacteria and hydrogenotrophic methanogens, which improves electron flow efficiency in bulk solutions away from electrodes.^17^ Nonelectroactive bacteria also respond variably to EFs; their metabolic activity and biofilm formation increase due to upregulated carbon metabolism and quorum-sensing genes, indirectly affecting electron transfer dynamics.^146^

Together, these population- and community-level responses translate multiscale microbial adaptations into measurable process-level outcomes, providing the critical link between fundamental EF microbe interactions and their practical implementation in wastewater treatment technologies.

## 8 Application of EF in Wastewater Treatment Plant

Representative studies demonstrating the effects of EF application on microbial activity, community composition, and process performance in wastewater and sludge matrices are compiled in Table 4. Examples of pilot-scale and full-scale wastewater treatment technologies employing EFs, together with their operational parameters and observed effects, are summarized in Table 5.

**Table 4:** Studies of EF Application in Wastewater Treatment and Effect on Microorganisms (Mixed Liquor Suspended Solids, MLSS; Current Density, CD; Chemical Oxygen Demand, COD; Soluble Chemical Oxygen Demand, SCOD)^a^

matrix	experimental conditions	effect on microorganisms	reference
wastewater	5; 10; 15; 20 A/m^2^, continuous; 24 h	5–15 A/m^2^ – positive impact on microorganisms (increased viable bacterial counts to ∼1.5 × 10^6^ CFU/mL at 5 A/m^2^) 20 A/m^2^ – decrease in bacterial viability (decline to ∼8 × 10^4^ CFU/mL)	^28^
lignin wastewater	0.03 A; continuous (12 h ON/12 h OFF; 2 h ON/2 h OFF)	enhanced microbial metabolic activity, better lignin removal (enriching lignin-degrading bacteria), increased dehydrogenase activity, increased Proteobacteria abundance (from 23.78% to 25.71–27.97%), ATP production increasing up to ∼2.07 μg/g MLSS	^147^
sewage sludge	3 A/m^2^; 7 A/m^2^; 15 days	lower CD (3 A/m^2^) supported higher microbial diversity, higher CD (7 A/m^2^) led to reduced diversity and earlier community stabilization, inhibition of sludge bulking, better sludge settleability	^148^
treated wastewater	0.55 V; continuous; 75 days	increase in microbial activity – high COD removal efficiency (94%), enhanced nitrogen removal (increased from 10.53 to 23.23% up to 56.88–86.80% removal efficiency)	^149^
sewage sludge (microplastic exposure)	0.6 V; 48 days	increase in microbial activity – mitigated the inhibitory effects of microplastics by reducing the decline in methane production (from −52.38% to −22.86% - with EF), improved SCOD removal efficiency (from −26.59% to −19.01%, with EF)	^150^
wastewater (microplastic exposure)	0.7 V; 48 days	increase in EPS production (from 265 to 289 mg/L to 280–341 mg/L), changes in microbial community – enriching *Firmicutes* (from 22.55 to 39.48%), *Bacteroidota* (from 12.68% to 26.99%), *Pseudomonadota* (from 0.35% to 10.66%)	^151^

a Electrical parameters are presented in the original units reported by the respective studies due to insufficient information for standardized unit conversion.

**Table 5:** Pilot-Scale and Industry Technologies Using EFs in Wastewater Treatment^a^

subject	experimental conditions	effect on microorganisms	reference
small-capacity wastewater treatment plant	5 V/m; 49.9 Hz; applied in biological treatment reactor	high microbial metabolic activity, faster denitrification and oxygen-consumption rates, reduced COD and total phosphorus	^18^
EF-coupled membrane bioreactor for phenol wastewater	30 V/m	enhanced phenol biodegradation and microbial metabolic activity, alleviated membrane fouling by lowering EPS production and improving sludge settleability	^98^
municipal wastewater	2 A/m^2^; during the log phase under light	enhanced microalgal biomass and lipid accumulation, accelerated nitrogen and phosphorus removal	^26^
urban wastewater treatment plant	3.0–3.8 × 10^6^ V/m; 40–200 μs	disinfection, 1.9–2.5 log reduction in resistant bacteria	^16^

a Electrical parameters are presented in the original units reported by the respective studies due to insufficient information for standardized unit conversion.

### 8.1 Synergistic Application of EFs with Wastewater Treatment Technologies

The synergistic application of electric fields with wastewater treatment technologies significantly enhances pollutant removal and alters the mechanisms between electrodes and microbial communities. Microbial removal of endocrine disruptors can be significantly enhanced by applying an electric field, as demonstrated in anaerobic electrochemical membrane bioreactors. These systems improve biodegradation rates of steroid estrogens like 17α-ethinylestradiol by providing supplemental energy to microbes, boosting microbial activity, electron transfer, and secretion of key enzymes, which convert them into less harmful small molecules.^152^ Bioelectrochemical systems coupling specific bacteria such as *Bacillus subtilis* with an applied voltage have also achieved high degradation efficiencies for bisphenol A, another common endocrine disruptor, under optimized conditions.^153^ Coupled systems combining biofilm electrode reactors with microbial fuel cells also achieve high antibiotic removal rates while suppressing antibiotic resistance genes, indicating that electric fields can reduce proliferation during treatment.^154^ PEF and electroporation techniques can inactivate antibiotic-resistant bacteria directly, enhancing antibiotic effectiveness and bacterial inactivation in wastewater.^155^ Electrocoagulation, an electrochemical process, effectively removes microplastics from wastewater with removal efficiencies often exceeding 90%, working through flocculation and charge neutralization rather than direct microbial degradation.^156^

Microbial adaptability to electric fields under extreme conditions, such as high salinity, low temperature, and high organic load, involves several mechanisms that enhance microbial activity, community structure, and pollutant degradation. Electrical stimulation can improve microbial salt tolerance by regulating ion transporters (Na^+^ and K^+^), promoting ATP production, and modifying EPS, which enhances bioactivity and biodiversity in a saline environment.^157^ The strength of the electric field critically affects microbial community structure and activity during electro-bioremediation of contaminated soils, with optimal voltages maximizing biomass and pollutant degradation.^158^ Additionally, microbes adapt to high organic loads in anaerobic digestion systems assisted by electric fields through the enrichment of electroactive bacteria and methanogens, improving methane yield even at ambient temperatures.^159^

The interface interaction mechanism between electrode materials and microbial communities primarily involves electron-transfer processes critical for microbial electrocatalysis and bioelectrochemical systems. Microorganisms transfer electrons to or from electrodes via direct contact through conductive pili or cytochromes or indirectly using soluble electron shuttles; this extracellular electron transfer is influenced by the electrode’s surface chemistry, topography, and material composition.^160^ Different electrode materials also shape the microbial community structure, with some materials favoring electroactive bacteria such as *Pseudomonadota* or denitrifiers that contribute to improved bioelectricity generation (Table 6).^161^

**Table 6:** Comparison of Different EF Technologies in Wastewater Treatment^a^

technology	treatment process	experimental conditions	effect	reference
EF-coupled micro/nano aeration biofilter	phosphorus removal	25V; 0.27 A; continuous	max. removal efficiency with EF 89.79% (control-45.75%)	^162^
electrocoagulation	microplastics removal	28.8–80.7 A/m^2^	removal efficiency 96.5%	^163^
microbial electrolysis cell	anaerobic digestion	1.4V; 1.8V	enhanced methanogen spp. growth, methane production enhanced 11.4–13.6 times	^164^
bioelectrochemical system	denitrification	0.9V	enhanced denitrification, nitrate removal efficiency 94.20%, enrich electroactive spp. *Pseudomonas* and *Arenimonas*	^165^
electro-dewatering	dewatering	5V;15V; 25V; 10 min	reduction of viable cells,	^166^
microbial electrolysis cell	heavy metals removal	0.9V	heavy metals removal efficiency 73.6–98.8%	^167^
electro algae-activated membrane bioreactor	denitrification	5 A/m^2^; continuous (5 min ON, 20 min OFF)	biomass growth, enhanced denitrification - NH_3_-N removal efficiency higher by 43.89%, COD reduction	^168^

a Electrical parameters are presented in the original units reported by the respective studies due to insufficient information for standardized unit conversion.

### 8.2 Comparison of EF Technologies with Conventional Wastewater Treatment Methods

Electric-field-based technologies represent one of several physical and electrochemical approaches used for wastewater treatment. Other commonly applied enhancement methods include ultrasound pretreatment, microwave irradiation, thermal conditioning, ozonation, and chemical conditioning. Although direct comparison between studies is difficult because of different experimental conditions, general advantages and disadvantages can still be identified. A summary of the main mechanisms, advantages, and limitations of these technologies is given in Table 7.

**Table 7:** Comparison of EF Technologies with Conventional Wastewater Treatment Methods^a^

technology	mechanism	advantages	limitations	reference
EF application	electroporation, microbial stimulation, electrooxidation, electron transfer	high efficiency, controllability, reduced chemical demand	energy demand, electrode corrosion, possible byproduct	^169–171^
ultrasound treatment	cavitation, radical formation	sludge disintegration, improved solubilization, enhanced biodegradability	high energy consumption, cavitation damage	^172–175^
microwave treatment	dielectric heating	fast heating, pathogen reduction, sludge solubilization	high energy input, expensive, incomplete understanding of nonthermal effects	^176–179^
chemical conditioning	coagulation, flocculation	better dewaterability, process simplicity, synergistic effect with oxidation	chemical consumption and cost, secondary chemical sludge production	^180–183^
ozonation	oxidative degradation via ROS,	biodegradability improvement, effective pollutant degradation	expensive, possible toxic byproducts	^184–186^
thermal treatment	heat-induced cell lysis and hydrolysis	high sludge solubilization, improved methane yield	high energy demand, expensive	^187–189^

a Cavitation is the formation, growth, and rapid collapse of vapor-filled bubbles (cavities) in a liquid.

## 9 Conclusions

This review demonstrates that EF application exerts profound effects on microorganisms in wastewater treatment systems, influencing cell membranes, cell walls, intracellular ion balance, oxidative stress responses, gene regulation, quorum sensing, and biofilm structure. Through these mechanisms, EFs can enhance pollutant degradation, sludge settleability and dewaterability, biofilm control, and bioenergy recovery. The effectiveness of EF-based approaches is highly dependent on field parameters and microbial cell structure, enabling both stimulation and inactivation of microbial activity under different operational conditions.

Compared with conventional wastewater treatment enhancement technologies, EF applications offer several advantages, including improved treatment efficiency, enhanced microbial activity, and reduced chemical consumption. A key advantage of EF applications is their versatility and potential for energy-efficient process intensification, particularly when low-voltage or low-frequency fields are applied. However, EF technologies also have several limitations. High-field intensities or prolonged exposure can increase energy demand, cause excessive sludge disintegration, and promote the release of EPS, negatively affecting process performance. In addition, electrode corrosion and electrochemical reactions at electrodes may generate toxic byproducts or accelerate material degradation, leading to increased maintenance requirements and potential environmental risks.

Future research should focus on specific and testable hypotheses derived from the observed EF microbial interactions in wastewater systems. For example, low-voltage EFs in the range of 0.5–1.1 V may selectively stimulate electroactive microorganisms and extracellular electron transfer, thereby enhancing denitrification, methane production, and microbial metabolic activity. Also, EFs around 10–80 V/m may promote EPS degradation and enrich EPS degrading microbial populations. However, moderate EF exposure (0.2 V) may stimulate adaptive EPS production in microorganisms such as Pseudomonas spp. Future studies should also determine the EF conditions under which QS activation shifts toward quorum quenching and oxidative degradation of signaling molecules, as both QS activation (0.5–0.8 V) and AHL degradation (5 A/m^2^) have been reported. The development of advanced electrode materials with improved corrosion resistance and long-term stability is also essential. In addition, further studies are needed to optimize the EF parameters for specific functional microbial communities involved in pollutant degradation and nutrient removal. Attention should be given to the application of EF technologies for the removal of emerging pollutants such as microplastics and antibiotic-resistant bacteria. Finally, pilot-scale and full-scale studies are required to evaluate the performance, energy efficiency, and operational feasibility of EF systems under real wastewater treatment conditions. With careful optimization, EF technologies hold significant promise for advancing sustainable and resilient wastewater treatment systems.
